# Does implantoplasty affect the failure strength of narrow and regular diameter implants? A laboratory study

**DOI:** 10.1007/s00784-020-03534-8

**Published:** 2020-09-07

**Authors:** Kristina Bertl, Flemming Isidor, Per Vult von Steyern, Andreas Stavropoulos

**Affiliations:** 1 Department of Periocdontology, Faculty of Odontology, University of Malmö, Malmö, Sweden; 2 Division of Oral Surgery, University Clinic of Dentistry, Medical University of Vienna, Vienna, Austria; 3 Section of Prosthetic Dentistry, Department of Dentistry, Aarhus University, Aarhus, Denmark; 4 Department of Dental Material Science and Technology, Faculty of Odontology, University of Malmö, Malmö, Sweden; 5 Division of Conservative Dentistry and Periodontology, University Clinic of Dentistry, Medical University of Vienna, Vienna, Austria; 6 Division of Regenerative Dental Medicine and Periodontology, CUMD University of Geneva, Geneva, Switzerland

**Keywords:** Implantoplasty, Dental implant, Implant failure, In vitro laboratory study, Mechanical complication, Dynamic loading

## Abstract

**Objective:**

To assess whether the impact of implantoplasty (IP) on the maximum implant failure strength depends on implant type/design, diameter, or material.

**Methods:**

Fourteen implants each of different type/design [bone (BL) and tissue level (TL)], diameter [narrow (3.3 mm) and regular (4.1 mm)], and material [titanium grade IV (Ti) and titanium-zirconium alloy (TiZr)] of one company were used. Half of the implants were subjected to IP in a computerized torn. All implants were subjected to dynamic loading prior to loading until failure to simulate regular mastication. Multiple linear regression analyses were performed with maximum implant failure strength as dependent variable and IP, implant type/design, diameter, and material as predictors.

**Results:**

Implants subjected to IP and TL implants showed statistically significant reduced implant failure strength irrespective of the diameter compared with implants without IP and BL implants, respectively. Implant material had a significant impact for TL implants and for regular diameter implants, with TiZr being stronger than Ti. During dynamic loading, 1 narrow Ti TL implant without IP, 4 narrow Ti TL implants subjected to IP, and 1 narrow TiZr TL implant subjected to IP were fractured.

**Conclusion:**

IP significantly reduced the maximum implant failure strength, irrespective implant type/design, diameter, or material, but the maximum implant failure strength of regular diameter implants and of narrow BL implants remained high.

**Clinical Relevance:**

IP seems to have no clinically relevant impact on the majority of cases, except from those of single narrow Ti TL implants, which may have an increased risk for mechanical complications. This should be considered for peri-implantitis treatment planning (e.g., communication of potential complications to the patient), but also in the planning of implant installation (e.g., choosing TiZr instead of Ti for narrow implants).

## Introduction

Overt peri-implantitis lesions regularly require a surgical intervention to achieve disease resolution [1, 2]. Depending on defect morphology and treatment approach, implantoplasty (IP), i.e., the mechanical removal of the implant threads and smoothening of the implant surface [3, 4], can be part of the surgical treatment protocol for implants with a rough surface. IP aims to achieve implant surface decontamination and also to reduce the risk of reinfection, and is recommended at those aspects of the implant, where bone healing and/or re-osseointegration is not expected. Although the clinical significance of IP (e.g., reduced bleeding indices and/or probing pocket depths, improved bone levels, etc.) has been confirmed only in a single randomized controlled clinical trial [3, 4], positive results have been reported in several case series (e.g., [5–12]), and IP appears as a widely used procedure.

Nevertheless, IP unavoidably causes a reduction of the implant mass, and thus it may weaken implant strength and increase implant fracture rate. A recent systematic review [13] summarized the available information on mechanical and/or biological complications due to IP. In 2 out of 3 laboratory studies identified [14–16], IP reduced implant strength; i.e., standard/regular diameter implants suffered up to 40% strength reduction [14, 16]. However, several other factors (e.g., implant type/design, implant material, etc.) may additionally affect implant strength after IP, but were not addressed in those studies.

Therefore, the present laboratory study aimed to assess whether the impact of IP on implant strength depends on implant type/design, diameter, and/or material.

## Material and methods

### Study design and implant material

**Fig. 1:**
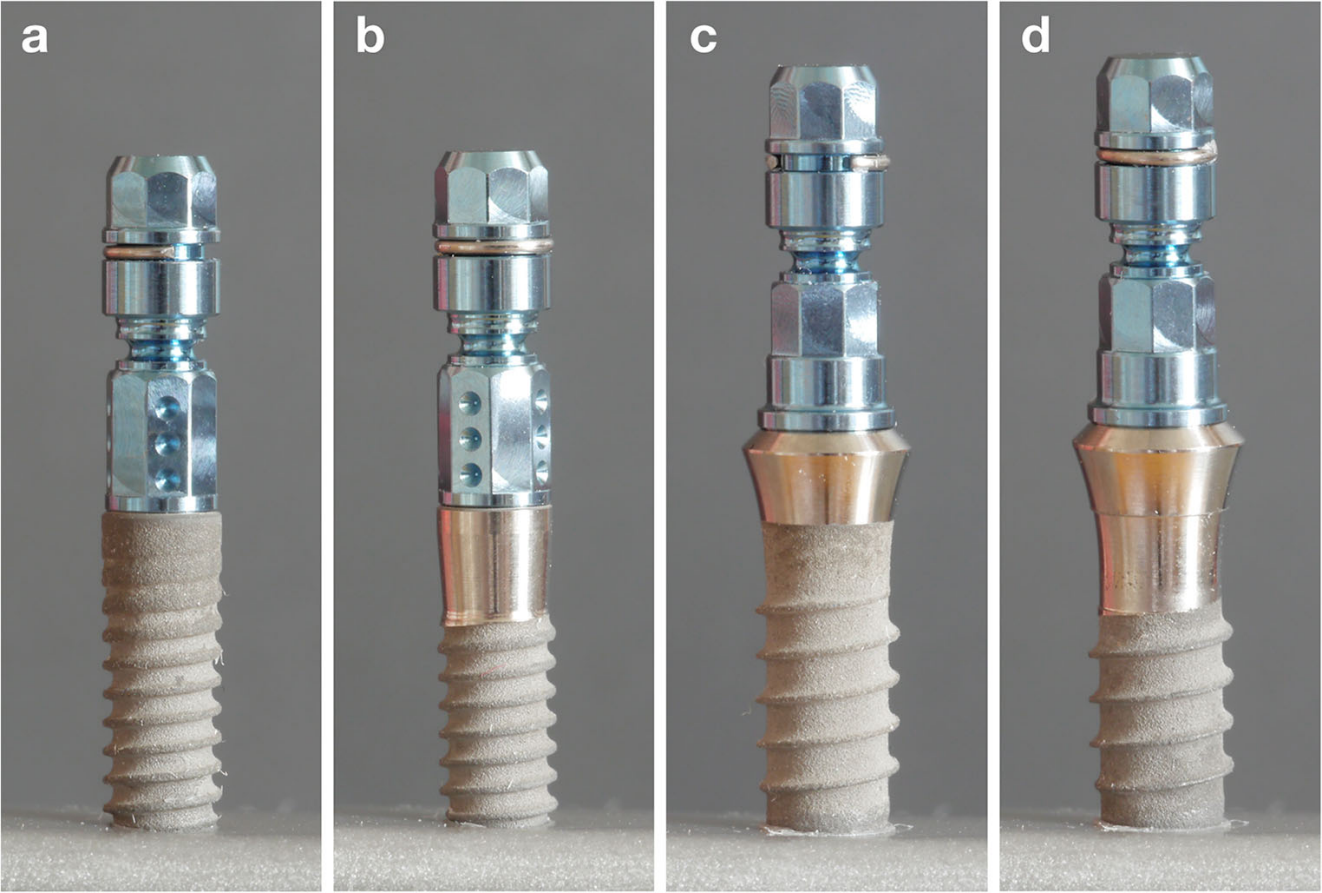
Implants of different type/design [(**a**, **b**) bone level, and (**c**, **d**) tissue level], diameter [(**a**, **b**) narrow—3.3 mm, and (**c**, **d**) regular—4.1 mm], and material [titanium (Ti) and titanium zirconium (TiZr) alloy] were tested with (**b**, **d**) or without (**a**, **c**) performing implantoplasty

All implants included herein were from one company (Institut Straumann AG, Basel, CH), had an internal connection type, and were 10 mm in length. Fourteen implants each of different type/design [bone level (BL) and tissue level (TL; Straumann Standard Plus)], diameter [narrow (3.3 mm) and regular (4.1 mm)], and material [titanium (Ti) and titanium zirconium (TiZr) alloy] were tested. Half of the implants were subjected to IP; i.e., the sample size of each final group was 7 implants based on a previous review, which recommends at least 6 specimens with identical test parameters for fracture strength analysis after fatigue testing [17]. Herein, 7 implants per group were included to compensate for any unforeseen issues during testing (Appendix 1)(Fig. 1).

### Implantoplasty

IP was performed with a computer-controlled torn (Tornos-Schaublin, 180-CCN - BL 3267, SCHAUBLIN MACHINES SA, Bévilard, CH) to ensure complete removal of the threads and the structured implant surface in a standardized fashion. Specifically, IP extended 3 mm apically from the implant neck in BL implants and from the machined/rough boundary in TL implants (Fig. 1). Depending on the implant type/design and diameter, the core diameter was reduced up to a maximum of 0.13 to 0.16 mm (i.e., narrow BL: 0.13 mm; narrow TL: 0.15 mm; regular BL: 0.14 mm; regular TL: 0.16 mm).

### Mechanical testing sequence

**Fig. 2:**
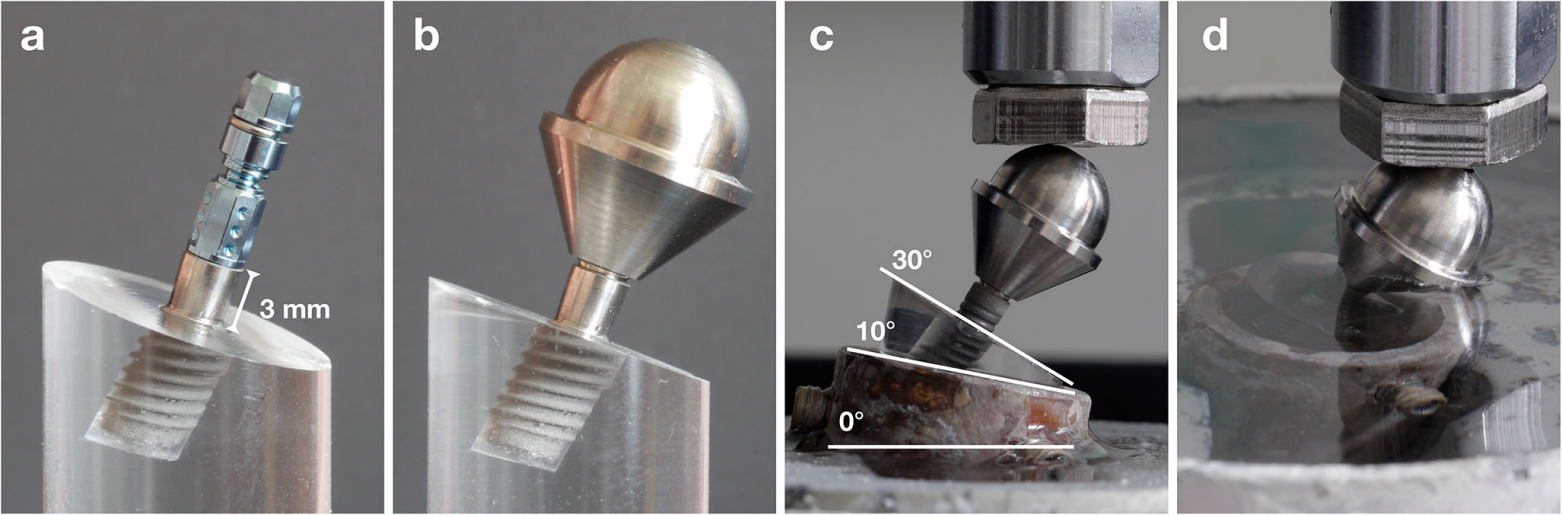
(**a**) The implants were inserted for 7 mm in poly-methyl-methacrylate block resulting in 3 mm exposed implant surface, and (**b**) furnished with hemispherical shaped, purpose-made abutments with the loading center located 11 mm from the “marginal bone level” of the implant. (**c**) The implants were loaded 30° off axis (10° by the metal holder plus 20° by the poly-methyl-methacrylate block) and (**d**) during the dynamic loading kept at room temperature in a moist environment (i.e., covered by water)

The mechanical testing of the implant material was performed according to DIN ISO 14801 (dentistry-fatigue test for endosseous implants, International Organization for Standardization). Specifically, the implants were inserted for 7 mm in poly-methyl-methacrylate block, resulting in 3-mm exposed rough implant surface (i.e., not including the 1.8-mm polished part of the TL implants) (Fig. 2a). This approach was chosen to simulate a horizontal marginal bone loss of 3 mm in both implant types even though it was resulting in a bigger lever for the TL implants (i.e., 3 mm of the rough implant surface plus 1.8 mm of the polished neck) compared with that of the BL implants. Implants were secured with a slow-curing transparent epoxy (EpoFix; Struers, Willich, Germany) resembling a bone-like environment with ≥ 3 GPa modulus of elasticity. All implants were furnished with hemispherical shaped, purpose-made abutments (Elos Medtech, Gørløse, Denmark) with the loading center located 11 mm from the “marginal bone level”" of the implant (Fig. 2b). The abutments were connected to the implant specimens with standardized force (35 Ncm) with a ratchet. Finally, the implants were installed 30° off axis (10° by the metal holder plus 20° by the poly-methyl-methacrylate block; Fig. 2c) for both the dynamic loading and loading until implant failure.

Prior to loading until failure, the implants were subjected to dynamic loading in a preload device (MTI Engineering AB, Lund, Sweden/Pamaco AB, Malmö, Sweden) to simulate mastication. Implants were loaded for 2,000,000 cycles at 2 Hz with 23 to 226 N at room temperature in a moist environment (i.e., covered by water; Fig. 2d). Dynamic loading force applied corresponded to 10 and 50% of the mean maximum failure strength of 3 narrow diameter Ti TL implants not subjected to IP.

**Fig. 3:**
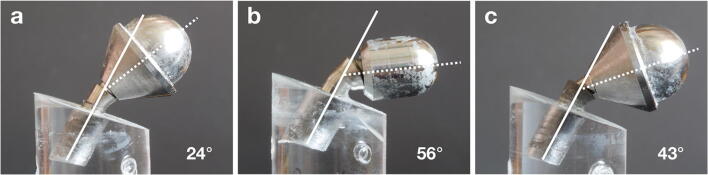
Implant failure was defined as (**a**) implant fracture or (**b**) and (**c**) severe deformation of the implant (i.e., bending of the implant and/or prosthetic component > 30°), whichever appeared first

Thereafter, implants were subjected to loading until failure in a universal testing machine (Instron 4465; Instron Co. Ltd, Norwood, MA, USA) with crosshead speed set at 1 mm/min. Maximum implant failure strength (N) was measured herein at timepoint of (1) implant fracture or (2) severe deformation of the implant (i.e., bending of the implant and/or prosthetic component > 30°), whichever appeared first (Fig. 3).

### Statistical analysis

Maximum implant failure strength (N) was defined as the primary outcome parameter. In a first step, a multiple linear regression analysis was performed for all implants with maximum implant failure strength as dependent variable and IP, implant type/design, diameter, and material as predictors. In a second step, multiple linear regression analyses were performed (1) separately for BL and for TL implants with maximum implant failure strength as dependent variable and IP, implant diameter, and material as predictors, and (2) separately for narrow and for regular diameter implants with maximum implant failure strength as dependent variable and IP, implant type/design, and material as predictors. Statistical analysis was performed using SPSS version 24.0 (SPSS Inc., Chicago, IL, USA) and *p* values < 0.05 were considered as statistically significant.

## Results

### Dynamic loading

None of the regular diameter implants and none of the narrow diameter BL implants were fractured during dynamic loading. However, 6 narrow diameter TL implants were fractured during dynamic loading. Specifically, the highest failure rate was present among the narrow diameter Ti TL implants subjected to IP, where 4 out of 7 implants were fractured; the remaining fractured implants were one narrow diameter Ti TL implant without IP and one narrow diameter TiZr TL implant subjected to IP.

### Loading until implant failure

**Fig. 4:**
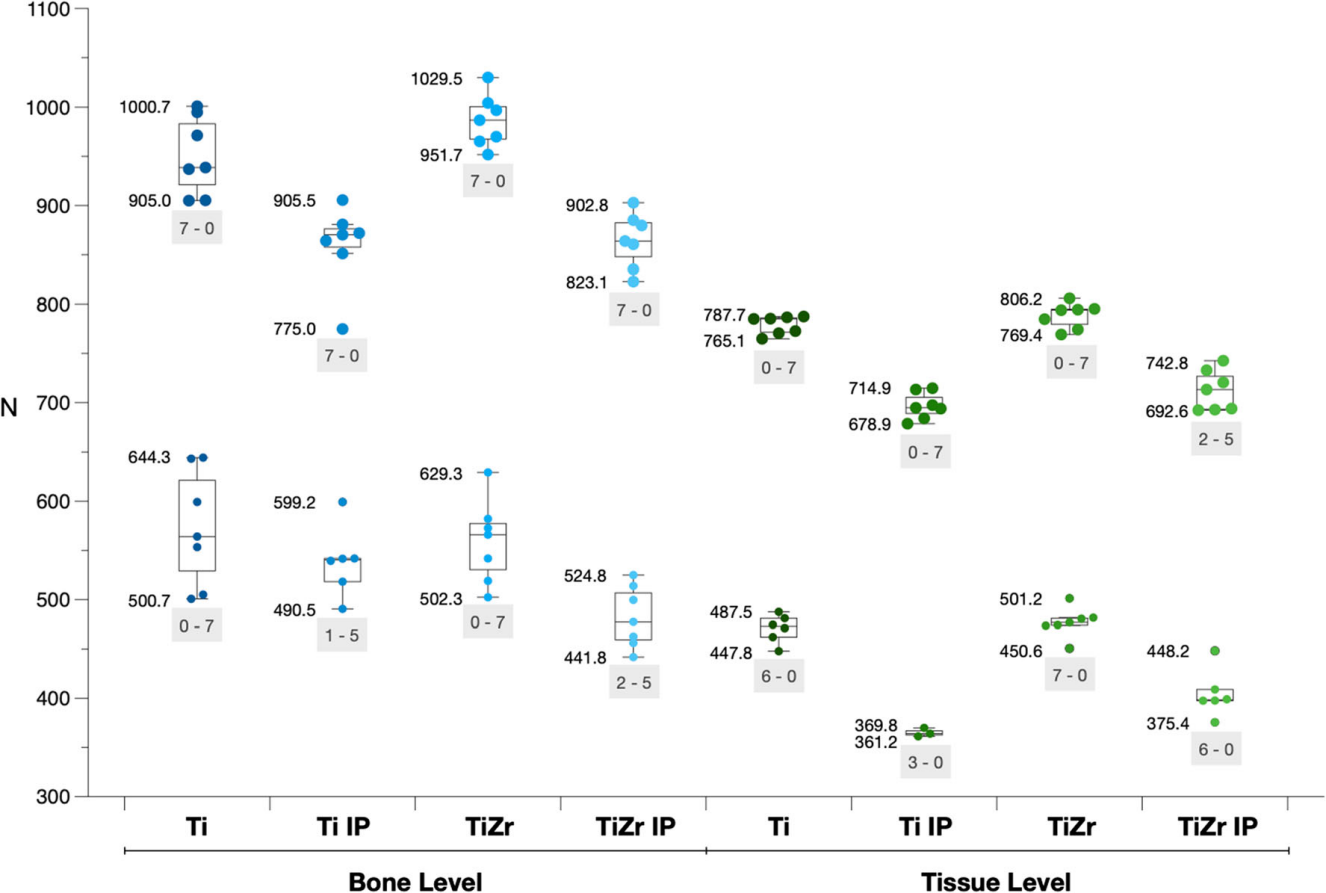
Results of the loading until implant failure tests (median and interquartile range; minimum and maximum value of each group is given to the left of the boxplots). Smaller points represent narrow diameter implants and bigger points regular diameter implants. The light grey text field indicates how many implants were fractured during loading until failure (first number) and how many bended > 30° before fracturing (second number). IP, implantoplasty; Ti, titanium alloy; TiZr, titanium zirconium alloy

Individual results of loading until failure are presented in Fig. 4 and Table 1. One narrow diameter Ti BL implant subjected to IP had to be excluded, due to a defect in the loading until failure test; i.e., only 6 implants were included in this specific group.

**Table 1:** Relative reduction (in %) of the maximum implant failure strength due to IP based on the median of each group

Groups			No implantoplasty	Implantoplasty	Relative reduction
			*Median (1.Q; 3.Q)*	*Median (1.Q; 3.Q)*	*%*
Narrow diameter	BL	Ti	564.03 (529.13; 621.22)	540.41 (518.13; 541.75)	**4.2**
		TiZr	565.91 (530.33; 577.32)	477.32 (459.34; 506.72)	**15.7**
	TL	Ti	472.76 (462.02; 481.08)	363.90 (362.56; 366.85)	**23.0**
		TiZr	476.78 (473.90; 481.08)	398.26 (397.45; 408.86)	**16.5**
Regular diameter	BL	Ti	938.53 (921.07; 982.90)	870.34 (857.72; 876.38)	**7.3**
		TiZr	986.58 (967.45; 1000.34)	863.90 (848.06; 882.43)	**12.4**
	TL	Ti	785.24 (771.82; 786.18)	695.04 (689.27; 705.64)	**11.5**
		TiZr	794.10 (779.74; 795.04)	713.56 (693.69; 726.98)	**10.1**

*1./3.Q, first/third quartile; BL, bone level implants; Ti, titanium alloy; TiZr, titanium zirconium alloy; TL, tissue level implants. Bold values indicate statistical significance*

The maximum implant failure strength of regular diameter BL and TL implants ranged from 775.0 to 1029.5 N and from 678.9 to 806.2 N, respectively, while the maximum implant failure strength of narrow diameter implants remained below 650 N (i.e., 441.8 to 644.3 N for narrow diameter BL and 361.2 to 501.2 N for narrow diameter TL implants). BL implants presented a higher range within each group (i.e., 78 to 144 N for the various BL implant groups) compared with TL implants (i.e., 9 to 73 N for the various TL implant groups); however, the range appeared unaffected by IP (Fig. 4). The relative reduction (in %) of the maximum implant failure strength due to IP, based on the median of each group, ranged from 4.2 to 23.0% with the narrow diameter implants, presenting a higher relative reduction in 3 out of 4 comparisons to the regular diameter implants (Table 1).

### Impact of IP, implant type/design, diameter, and/or material on implant strength

Multiple linear regression analysis, including all implants, revealed IP, implant type/design, and diameter as statistically significant predictors (*p* < 0.001; Table 2). Specifically, implants subjected to IP and TL implants presented a statistically significant lower maximum implant failure strength compared with implants without IP and BL implants, respectively, while regular diameter implants presented a statistically significant higher maximum implant failure strength compared with narrow diameter implants. Separate multiple linear regression analyses were also performed for (1) BL and TL implants (Table 3) and (2) narrow and regular diameter implants (Table 4). Both, BL and TL implants, implants subjected to IP and narrow diameter implants presented a statistically significant lower maximum implant failure strength (*p* < 0.001) compared with implants without IP and regular diameter implants, respectively. Yet, implant material had a statistically significant impact only among TL implants, with TiZr being stronger than Ti (*p* = 0.002) (Table 3). Regarding narrow and regular diameter implants, for both diameters, implants subjected to IP and TL implants presented a statistically significant lower maximum implant failure strength (*p* < 0.001) compared with implants without IP and BL implants, respectively. Yet, implant material showed a statistically significant impact only among regular diameter implants, with TiZr being stronger than Ti (*p* = 0.027) (Table 4).

**Table 2:** Multiple linear regression analysis with maximum implant failure strength (N) as dependent variable and implantoplasty, implant type/design, diameter, and material as predictors

Parameter		Coefficient	95% Confidence interval		*p* value
			*Lower*	*Upper*	
Implantoplasty	*No*	0.0			**< 0.001**
	*Yes*	− 82.90	− 97.40	− 68.40	
Implant type/design	*Bone level*	0.0			**< 0.001**
	*Tissue level*	− 139.96	− 154.46	− 125.46	
Implant diameter	*Narrow*	0.0			**< 0.001**
	*Regular*	347.28	332.74	361.82	
Implant material	*Ti*	0.0			0.473
	*TiZr*	5.27	− 9.23	19.77	

*Ti (titanium) and TiZr (titanium zirconium) alloy. Bold values indicate statistical significance*

**Table 3:** Multiple linear regression analyses separately for BL and TL implants with maximum implant failure strength (N) as dependent variable and implantoplasty, implant diameter, and material as predictors.

Parameter		Coefficient	95% Confidence interval		*p* value
			*Lower*	*Upper*	
Bone level implants					
Implantoplasty	*No*	0.0			**< 0.001**
	*Yes*	− 82.26	− 105.69	− 58.83	
Implant diameter	*Narrow*	0.0			**< 0.001**
	*Regular*	378.50	355.07	401.93	
Implant material	*Ti*	0.0			0.612
	*TiZr*	− 5.96	− 29.39	17.47	
Tissue level implants					
Implantoplasty	*No*	0.0			**< 0.001**
	*Yes*	− 81.39	− 90.70	− 72.09	
Implant diameter	*Narrow*	0.0			**< 0.001**
	*Regular*	312.97	303.60	322.34	
Implant material	*Ti*	0.0			**0.002**
	*TiZr*	15.16	5.85	24.46	

*Ti (titanium) and TiZr (titanium zirconium) alloy. Bold values indicate statistical significance*

**Table 4:** Multiple linear regression analyses separately for narrow and regular diameter implants with maximum implant failure strength (N) as dependent variable and implantoplasty, implant type/design, and material as predictors

Parameter		Coefficient	95% Confidence interval		*p* value
			*Lower*	*Upper*	
Narrow diameter implants					
Implantoplasty	*No*	0.0			**< 0.001**
	*Yes*	− 67.87	− 89.77	− 45.97	
Implant type/design	*Bone level*	0.0			**< 0.001**
	*Tissue level*	− 102.02	− 123.92	− 80.11	
Implant material	*Ti*	0.0			0.298
	*TiZr*	− 11.45	− 33.35	10.45	
Regular diameter implants					
Implantoplasty	*No*	0.0			**< 0.001**
	*Yes*	− 92.49	− 107.00	− 77.99	
Implant type/design	*Bone level*	0.0			**< 0.001**
	*Tissue level*	− 170.83	− 185.33	− 156.32	
Implant material	*Ti*	0.0			**0.027**
	*TiZr*	16.42	1.92	30.93	

*Ti (titanium) and TiZr (titanium zirconium) alloy. Bold values indicate statistical significance*

### Failure type

**Fig. 5:**
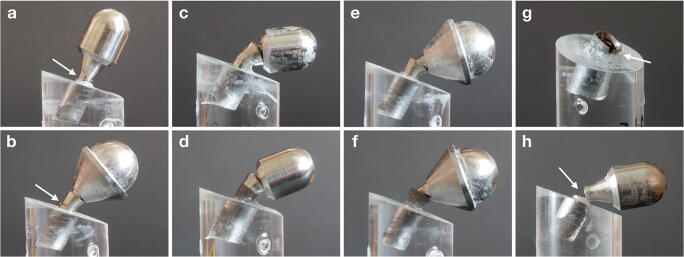
Collection of various implant failures; (**a**) and (**b**) implant fracture at the implant body during loading until failure, (**c–f**) severe deformation of the implant during loading until failure (i.e., bending of the implant and/or prosthetic component >30°); (**g**) implant fracture at the implant neck during loading until failure; and (**h**) implant fracture during dynamic loading

Failure type (i.e., implant fracture or bending of the implant and/or prosthetic component > 30°) was recorded for each implant (Figs. 4 and 5). All narrow diameter TL implants and all regular diameter BL implants were fractured before bending > 30° irrespective whether IP was performed or not. All narrow diameter BL implants without IP and all regular diameter TL implants without IP bended > 30° before fracturing, while a few cases from these groups subjected to IP were fractured instead of bending > 30° (i.e., narrow diameter Ti BL implants: 1 case; narrow diameter TiZr BL implants: 2 cases; regular diameter Ti TL implants: 0 cases; regular diameter TiZr TL implants: 2 cases). Interestingly, only 2 regular Ti BL implants without IP were fractured at the neck, while all other fractures occurred at the implant body.

## Discussion

Peri-implantitis treatment requires in most cases a surgical approach to get access to the implant surface for decontamination. One approach for implants with a rough surface includes removal of the implant threads and smoothening of the implant surface (i.e., implantoplasty, IP) at the aspects of the implant, where bone healing and/or re-osseointegration is not expected. Since IP unavoidably causes a reduction of the implant mass, it may also weaken the implant and lead to implant fracture. The present laboratory study confirmed that IP causes statistically significant reduction of the maximum implant failure strength, irrespective implant type/design, diameter, and material. Up to now 4 laboratory studies [14–16, 18] and one finite element analysis [19] are available on this topic, describing that the impact of IP on implant failure strength appeared to depend on the implant diameter and connection type. Specifically, while wide diameter implants (i.e., 4.7 mm diameter) were not significantly affected by IP [14], contradicting results were reported for regular diameter implants (i.e., 3.75 to 4.3 mm diameter), with 2 studies [15, 18] showing no significant impact of IP on the fracture strength of regular diameter implants, and 2 studies demonstrating statistically significant reduction (up to 40%) in implant failure strength [14, 16]. Further, reduction in fracture strength varied among connection types, with a Morse taper connection being least affected [16]. Herein, IP resulted in significant reduction in implant failure strength in both standard and narrow diameter implants. However, IP seemed to affect more the narrow diameter implants than standard diameter implants, and implant type/design was also shown as a relevant parameter. In contrast to previous data [16], the range of the maximum implant failure strength among the implants within each specific group appeared unaffected by IP; i.e., herein, the range did not increase relevantly in implants subjected to IP. The fact that TL implants are weaker than BL implants is at least partly explained by the fact that TL implants were exposed (i.e., out of the plexiglass holder) at a larger extent compared to BL implants; i.e., 3 mm of the rough surface plus 1.8 mm of the TL neck, thus resulting in a bigger lever. Although previous studies [20, 21] had already indicated that a higher “marginal bone loss” further reduces implant strength, this approach was chosen to simulate a similar amount of horizontal marginal bone loss in both implant types.

In the present study, the impact of IP on narrow diameter implants (i.e., ≤ 3.5 mm) was assessed for the first time. It appears obvious that a smaller diameter implant, which has a thinner metal wall compared to regular/standard diameter implants, would also be more affected from IP. Indeed, fractures during dynamic loading occurred only among narrow diameter TL implants (i.e., 5 out 6 fractures), mainly those subjected to IP. In this context, fracture rate during dynamic loading was clearly lower in narrow TiZr implants compared with narrow Ti implants; i.e., only a single TiZr implant subjected to IP fractured vs. 5 Ti implants. Indeed, the results of the separate multiple linear regression analyses showed that TiZr implants had a statistically significant increased maximum implant failure strength compared with Ti implants, among TL implants and among regular diameter implants. The lack of statistical significance among the narrow diameter implants is most likely due to the high “drop-out rate” among the narrow Ti TL implants subjected to IP. However, the higher fracture rate among narrow Ti TL implants subjected to IP compared with that of narrow TiZr TL implants subjected to IP (i.e., 4 vs. 1 fractures, respectively), in combination with the fact that the highest maximum load value of the narrow Ti TL implants subjected to IP was lower than the lowest maximum load value of the narrow TiZr TL implants subjected to IP, gives a strong indication for an effect of the material also among the narrow implants. Previous laboratory studies have indeed indicated a higher strength of TiZr compared with Ti implants (for overview see: [22]); however, the clinical relevance of these reports is yet unknown. A recent systematic review [23], assessing the clinical performance of narrow diameter Ti and TiZr implants, showed that similar success rates in terms of survival and marginal bone loss, independent of the region in the mouth, are obtained from both types of implants at least on the short-term.

The results herein showed that despite the fact that IP resulted in a statistically significant reduction of the maximum implant failure strength, the forces required to fracture or deformate all regular diameter implants and narrow BL implants remained high (i.e., > 650 and 440 N, respectively). Forces occurring in the natural dentition during regular mastication range between 100 and 300 N [24]. Single implants as well as implant-supported fixed bridges appear to be loaded with similar or slightly lower forces [25–27], while loading forces decrease in implant-supported cross-arch restorations, and even more in implant-supported overdentures [28–30]. Indeed, no study/case report describing implant fracture after IP was identified in a recent systematic review on mechanical and/or biological complications due to IP [13]. Nevertheless, direct comparison of forces derived from laboratory studies to those from clinical studies should be made with care, due to limitations such as differences in the loading mechanism (i.e., only vertical forces in the laboratory vs. a combination of vertical and horizontal loading forces in the mouth) or in the superstructure geometry (i.e., standardized hemispherical shaped, purpose-made abutments in the laboratory vs. anatomically shaped crowns in the mouth) [31].

The present study shows some important differences/advantages in terms of study design compared with previous laboratory studies on IP. In contrast to previous studies, all implants herein were subjected to dynamic loading prior to loading until failure, to simulate regular mastication and add a certain “aging effect” on the implants [17]. The implants were loaded 2,000,000 cycles, which correspond to the masticatory activity of a couple of years, and the forces applied were within the range of regular chewing forces (i.e., up to 300 N) [24]. Further, IP was performed with a computer-controlled torn, instead of “free hand,” which was used in most of the previous studies [14, 15, 18], to ensure removal of a standardized amount of implant material. Although this approach does not represent the true clinical situation, it ensured that exactly the same amount of metal was removed from every single implant of the various groups. Next, in the present study, a horizontal bone loss of 3 mm was simulated in contrast with the previous laboratory studies simulating 5 to 6 mm bone loss. Thus, the results of the present study may be applicable only in cases of incipient to moderate peri-implantitis (i.e., about 3 mm of bone loss) than to advanced (i.e., ≥ 5 mm of bone loss) peri-implantitis cases. Finally, herein, implant failure was defined as (1) implant fracture or (2) severe deformation of the implant (i.e., bending of the implant and/or prosthetic component > 30°), whichever appeared first. It appeared reasonable that an implant, which is already bended beyond 30°, should be considered failure although fracture may occur only at a later timepoint.

In conclusion, within this laboratory setting, IP significantly reduced maximum implant failure strength, irrespective implant type/design, diameter, or material. However, the maximum implant failure strength of regular diameter and narrow BL implants remained high despite IP (i.e., > 650 and 440 N, respectively), while > 50% of the narrow Ti TL implants subjected to IP were fractured already during dynamic loading, simulating regular mastication. Thus, IP seems to have no clinically relevant impact on the majority of cases, except from those of single narrow Ti TL implants, which may have an increased risk for mechanical complications. The latter should be considered for peri-implantitis treatment planning (e.g., communication of potential complications to the patient), but also in the planning of implant installation (e.g., choosing TiZr instead of Ti for narrow implants).

## References

[1] Klinge B, Klinge A, Bertl K, Stavropoulos A (2018). Peri-implant diseases. Eur J Oral Sci.

[2] Renvert S, Polyzois I (2018, 2000) Treatment of pathologic peri-implant pockets. Periodontol 76:180–190. 10.1111/prd.12149 10.1111/prd.12149 29239086

[3] Romeo E, Ghisolfi M, Murgolo N, Chiapasco M, Lops D, Vogel G (2005). Therapy of peri-implantitis with resective surgery. A 3-year clinical trial on rough screw-shaped oral implants. Part I: clinical outcome. Clin Oral Implants Res.

[4] Romeo E, Lops D, Chiapasco M, Ghisolfi M, Vogel G (2007). Therapy of peri-implantitis with resective surgery. A 3-year clinical trial on rough screw-shaped oral implants. Part II: radiographic outcome. Clin Oral Implants Res.

[5] Bianchini MA, Galarraga-Vinueza ME, Apaza-Bedoya K, De Souza JM, Magini R, Schwarz F (2019). Two to six-year disease resolution and marginal bone stability rates of a modified resective-implantoplasty therapy in 32 peri-implantitis cases. Clin Implant Dent Relat Res.

[6] Bianchini MA, Galarraga-Vinueza ME, Bedoya KA, Correa BB, de Souza MR, Schwarz F (2020). Implantoplasty Enhancing Peri-implant Bone Stability Over a 3-Year Follow-up: A Case Series. Int J Periodontics Restorative Dent.

[7] Matarasso S, Iorio Siciliano V, Aglietta M, Andreuccetti G, Salvi GE (2014). Clinical and radiographic outcomes of a combined resective and regenerative approach in the treatment of peri-implantitis: a prospective case series. Clin Oral Implants Res.

[8] Pommer B, Haas R, Mailath-Pokorny G, Fürhauser R, Watzek G, Busenlechner D, Müller-Kern M, Kloodt C (2016). Periimplantitis treatment: long-term comparison of laser decontamination and implantoplasty surgery. Implant Dent.

[9] Schwarz F, Sahm N, Iglhaut G, Becker J (2011). Impact of the method of surface debridement and decontamination on the clinical outcome following combined surgical therapy of peri-implantitis: a randomized controlled clinical study. J Clin Periodontol.

[10] Schwarz F, John G, Mainusch S, Sahm N, Becker J (2012). Combined surgical therapy of peri-implantitis evaluating two methods of surface debridement and decontamination. A two-year clinical follow up report. J Clin Periodontol.

[11] Schwarz F, Hegewald A, John G, Sahm N, Becker J (2013). Four-year follow-up of combined surgical therapy of advanced peri-implantitis evaluating two methods of surface decontamination. J Clin Periodontol.

[12] Schwarz F, John G, Schmucker A, Sahm N, Becker J (2017). Combined surgical therapy of advanced peri-implantitis evaluating two methods of surface decontamination: a 7-year follow-up observation. J Clin Periodontol.

[13] Stavropoulos A, Bertl K, Eren S, Gotfredsen K (2019). Mechanical and biological complications after implantoplasty - A systematic review. Clin Oral Implants Res.

[14] Chan HL, Oh WS, Ong HS (2013). Impact of implantoplasty on strength of the implant-abutment complex. Int J Oral Maxillofac Implants.

[15] Costa-Berenguer X, García-García M, Sánchez-Torres A, Sanz-Alonso M, Figueiredo R, Valmaseda-Castellón E (2018). Effect of implantoplasty on fracture resistance and surface roughness of standard diameter dental implants. Clin Oral Implants Res.

[16] Gehrke SA, Aramburú Júnior JS, Dedavid BA, Shibli JA (2016). Analysis of implant strength after implantoplasty in three implant-abutment connection designs: an in vitro study. Int J Oral Maxillofac Implants.

[17] Coray R, Zeltner M, Özcan M (2016). Fracture strength of implant abutments after fatigue testing: A systematic review and a meta-analysis. J Mech Behav Biomed Mater.

[18] Sahrmann P, Luso S, Mueller C (2019). Titanium implant characteristics after implantoplasty: an in vitro study on two different kinds of instrumentation. Int J Oral Maxillofac Implants.

[19] Tribst JPM, Dal Piva AMO, Shibli JA, Borges ALS, Tango RN (2017). Influence of implantoplasty on stress distribution of exposed implants at different bone insertion levels. Braz Oral Res.

[20] Gehrke SA, Souza Dos Santos Vianna M, Dedavid BA (2014). Influence of bone insertion level of the implant on the fracture strength of different connection designs: an in vitro study. Clin Oral Investig.

[21] Suzuki H, Hata Y, Watanabe F (2015). Implant fracture under dynamic fatigue loading: influence of embedded angle and depth of implant. Odontology.

[22] Grandin HM, Berner S, Dard M (2012). A review of titanium zirconium (TiZr) alloys for use in endosseous dental implants. Materials.

[23] Iegami CM, Uehara PN, Sesma N, Pannuti CM, Tortamano Neto P, Mukai MK (2017). Survival rate of titanium-zirconium narrow diameter dental implants versus commercially pure titanium narrow diameter dental implants: A systematic review. Clin Implant Dent Relat Res.

[24] Vallittu PK, Könönen M (2013) Biomechanical aspects and material properties. A Textbook of Fixed Prosthodontics: The Scandinavian Approach:116–130.

[25] Al-Omiri MK, Sghaireen MG, Alhijawi MM, Alzoubi IA, Lynch CD, Lynch E (2014). Maximum bite force following unilateral implant-supported prosthetic treatment: within-subject comparison to opposite dentate side. J Oral Rehabil.

[26] Goshima K, Lexner MO, Thomsen CE, Miura H, Gotfredsen K, Bakke M (2010). Functional aspects of treatment with implant-supported single crowns: a quality control study in subjects with tooth agenesis. Clin Oral Implants Res.

[27] Meena A, Jain V, Singh N, Arora N, Jha R (2014). Effect of implant-supported prosthesis on the bite force and masticatory efficiency in subjects with shortened dental arches. J Oral Rehabil.

[28] Luraschi J, Schimmel M, Bernard JP, Gallucci GO, Belser U, Müller F (2012). Mechanosensation and maximum bite force in edentulous patients rehabilitated with bimaxillary implant-supported fixed dental prostheses. Clin Oral Implants Res.

[29] Müller F, Hernandez M, Grütter L, Aracil-Kessler L, Weingart D, Schimmel M (2012). Masseter muscle thickness, chewing efficiency and bite force in edentulous patients with fixed and removable implant-supported prostheses: a cross-sectional multicenter study. Clin Oral Implants Res.

[30] van der Bilt A, Burgers M, van Kampen FM, Cune MS (2010). Mandibular implant-supported overdentures and oral function. Clin Oral Implants Res.

[31] Geringer A, Diebels S, Nothdurft FP (2014). Influence of superstructure geometry on the mechanical behavior of zirconia implant abutments: a finite element analysis. Biomed Tech (Berl).

